# Polydimethylsiloxane-Zinc Oxide Nanorod-Based Triboelectric Nanogenerator for Compression Applications

**DOI:** 10.3390/ma18071392

**Published:** 2025-03-21

**Authors:** Shiyu Zhao, Guanghui Han, Huaxia Deng, Mengchao Ma, Xiang Zhong

**Affiliations:** 1 School of Instrument Science and Opto-Electronics Engineering, Hefei University of Technology, Hefei 230009, China; syzhao@mail.hfut.edu.cn (S.Z.); hgh331@mail.hfut.edu.cn (G.H.); mmchao@hfut.edu.cn (M.M.); 2 CAS Key Laboratory of Mechanical Behavior and Design of Materials, Department of Modern Mechanics, University of Science and Technology of China, Hefei 230027, China

**Keywords:** triboelectric nanogenerator, ZnO nanorods, energy harvesting

## Abstract

In this study, to enhance the output performance of a contact-separation mode triboelectric nanogenerator (TENG), a zinc oxide nanorod (ZnO NR) film with piezoelectric properties was integrated into a Polydimethylsiloxane (PDMS) film as the dielectric layer. The working mechanism of the PDMS-ZnO NR-based TENG was theoretically analyzed in two stages: charge transfer during contact electrification on the material surface and charge movement in the electrostatic induction process. The output characteristics of the PDMS-ZnO NR-based TENG were investigated and compared with those of a PDMS-based TENG. The experimental results demonstrate that the PDMS-ZnO NR-based TENG reached an open-circuit voltage of 39.34 V, representing an increase of 64.5% compared to the PDMS-based TENG. The maximum output power of a 4 cm × 4 cm PDMS-ZnO NR-based TENG reached 82.2 μW. Using a specially designed energy-harvesting circuit, the generated electrical energy was stored in a capacitor, which was charged to 1.47 V within 1 min and reached 3 V in just 2.78 min. This voltage was sufficient to power over 20 LEDs and small sensors. Additionally, the TENG was integrated into the sole of footwear, where the electrical signals generated by compression could be utilized for step counting and gait analysis.

## 1. Introduction

With the advancement of technology and the improvements in living standards, electronic devices have become increasingly prevalent in daily life [1]. These devices typically operate through integrated wiring systems. However, as the number of devices grows, the wiring can become excessively complex, making installation and maintenance more challenging. Conversely, standalone electronic devices powered by batteries require frequent battery replacements, which increases the risk of power failure. To address these challenges, clean energy sources, such as wind, solar, tidal, and biomass energy, have been explored for powering distributed devices [2]. However, these energy-harvesting methods share a common limitation: the required equipment is typically large, and the energy output is substantial, making them unsuitable for small-scale, distributed energy harvesting in everyday applications.

Triboelectric nanogenerators (TENGs) function as an energy-harvesting technology, operating based on the coupled effects of contact electrification and electrostatic induction [3,4]. TENGs offer a wide range of materials for triboelectricity and support various operational modes. Additionally, studies have demonstrated that TENGs possess several advantages, including fast response, small size, simple structure, high sensing precision, low cost, and lightweight design [5]. Compared to electromagnetic generators, TENGs exhibit superior low-frequency response, making them highly effective in converting small-scale, low-frequency energy [6]. Contact-separation mode TENGs generate electricity by utilizing charge transfer that occurs when materials with different triboelectric polarities come into contact and then separate [7]. This type of TENG is commonly used for energy harvesting from vertical motion, such as in applications involving compression and vibration. In contrast, lateral-sliding mode TENGs operate in a parallel motion direction and are typically employed for harvesting energy from relative sliding and reciprocating movements [8], such as the interaction between rotating wheels and brakes or friction between rotating discs. Compared to the contact-separation and lateral-sliding modes, single-electrode mode TENGs have a simpler structure, requiring only one triboelectric layer connected to an electrode [9]. Due to this structural simplicity, the single-electrode mode is often used in marine, river, and waterway environments for energy harvesting and water level detection [10]. Furthermore, single-electrode TENGs can be embedded in clothing or even adhered to the skin to collect electrical signals [11,12]. Freestanding triboelectric layer TENGs introduce an independent triboelectric layer that guides charge transfer between adjacent electrodes, thereby expanding the range of applicable motions [13]. This type of TENG is frequently applied in sensing and measurement technologies [14].

TENGs primarily harvest low-frequency, small-scale triboelectric energy. Although they exhibit higher efficiency compared to electromagnetic generators under similar conditions, their overall power output remains relatively low. To enhance power generation performance and expand the potential applications of these devices, extensive research has been conducted. This includes efforts to increase energy output by optimizing the overall structural design [15], modifying the surface microstructure of materials [16], and employing composite materials [17]. Zinc oxide (ZnO), as a transition metal oxide, possesses several unique properties that make it highly suitable for triboelectric and piezoelectric energy harvesting [18,19]. Its non-centrosymmetric wurtzite structure imparts piezoelectric properties, enabling charge separation on the surface of ZnO crystals when subjected to mechanical stress [20]. The nanostructured form of ZnO, with its high specific surface area, enhances both triboelectric and piezoelectric effects [21]. Therefore, the ZnO nanorods (NRs) encapsulated in PDMS and connected to electrodes can form a piezoelectric nanogenerator (PENG) that converts mechanical pressure into electrical energy [22]. Additionally, ZnO NRs can also function directly as a dielectric layer to create a contact-separation TENG [23]. Through theoretical and experimental analyses, it has been demonstrated that this configuration offers superior anti-reflective properties and faster response times compared to conventional ITO-PET films. Moreover, ZnO NRs have also been embedded within the dielectric layer, either parallel or perpendicular to the electrode surface [24,25]. These structures function by releasing charges during the contact and compression process, thereby increasing the number of free electrons in the material and ultimately enhancing the energy output of TENGs.

In this work, the piezoelectric properties of ZnO nanorods (NRs) were leveraged by integrating them into PDMS as the dielectric layer to enhance the electrical output of a TENG. The enhancement effect of ZnO NRs on TENG power generation was demonstrated through both theoretical analysis and experimental validation. The output characteristics of the PDMS-ZnO NR-based TENG were tested and compared with those of a PDMS-based TENG. Subsequently, charging and discharging performance, as well as long-term stability, were evaluated. Finally, the TENG was integrated into footwear to harvest energy and sense during walking or running. This approach not only provides continuous power for wearable electronic devices but also enables applications such as gait analysis and health monitoring, aligning with the development trend of smart wearables.

## 2. Materials and Methods

### 2.1. Materials

Hexamethyltetramine (HMTA), zinc nitrate hexahydrate($\left[\mathrm{Zn}{\left({\mathrm{NO}}_{3}\right)}_{2}.6H_{2}O\right]$), and ammonia solution were purchased from Bida Environmental Technology Co., Ltd. (Shenzhen, China). Ethanol was purchased from Xilong Scientific Co., Ltd. (Shantou, China). Polydimethylsiloxane(SYLGARD184, PDMS) was purchased from DOWSIL Corporation (Midland, TX, USA). PET-ITO conductive film was purchased from Siyu Industrial Products Co., Ltd. (Shenzhen, China). Deionized water was used throughout the experiment.

### 2.2. Fabrication of PDMS-ZnO NR-Based TENG

**Figure 1:**
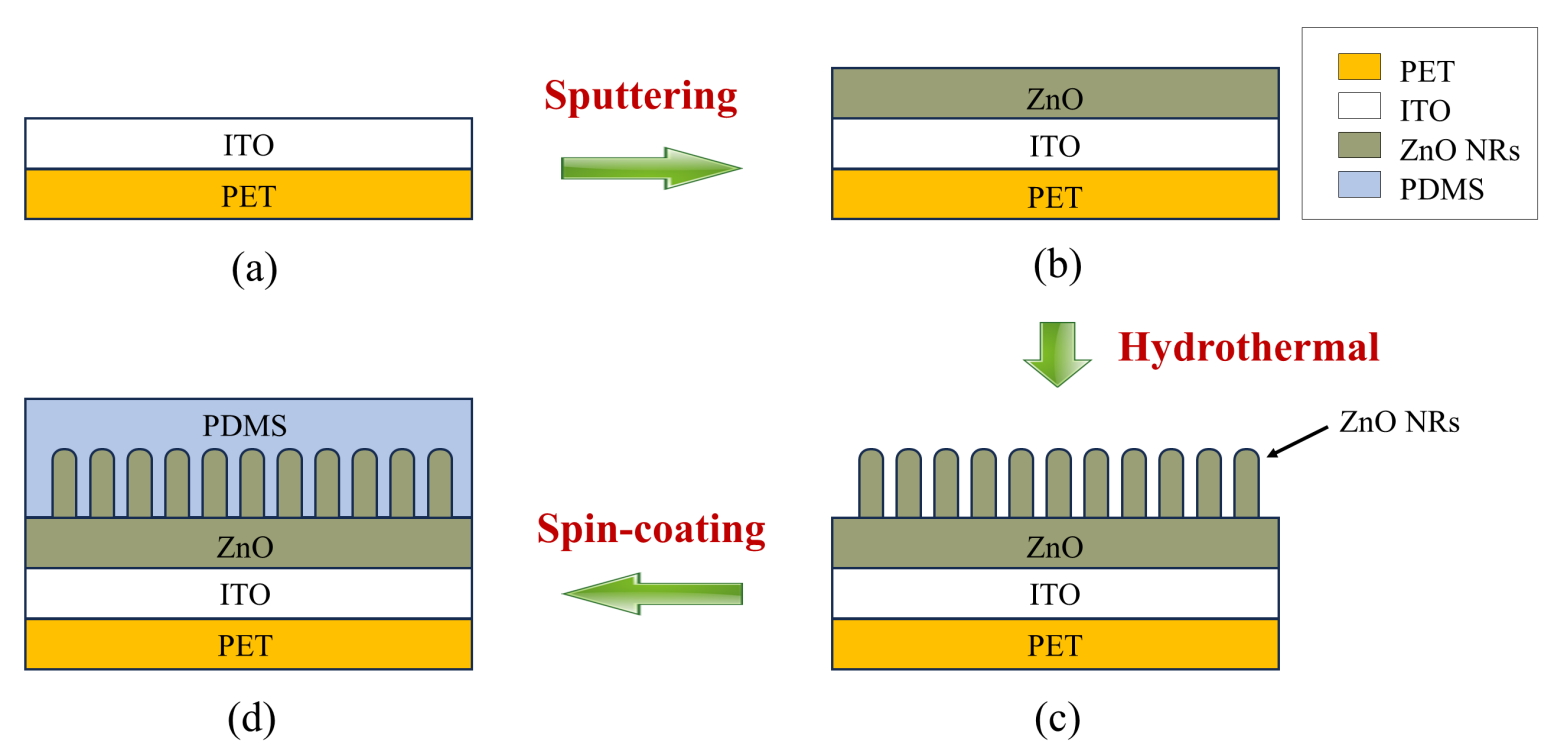
Fabrication process of the PDMS-ZnO NR triboelectric layer. (**a**) PET-ITO substrate; (**b**) ZnO seed layer deposited by magnetron sputtering; (**c**) ZnO NRs grown via hydrothermal method; (**d**) PDMS coated by spin coating.

**Figure 2:**
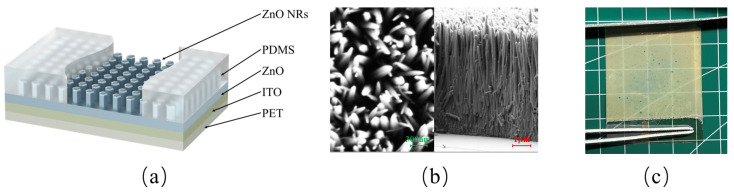
Schematic of the PDMS-ZnO NR film. (**a**) Structural diagram; (**b**) SEM image of the ZnO NRs; (**c**) photograph of the film.

The TENG used in this study consists of two electrodes. Aluminum, a metal prone to electron loss, was chosen as the positive electrode, which also serves as the triboelectric layer. The negative electrode is composed of a composite of PDMS and ZnO NRs, which exhibit opposite polarity. The fabrication process is illustrated in Figure 1. (I) A PET substrate coated with an ITO conductive film serves as the current carrier. (II) A 100 nm thick ZnO seed layer was deposited onto the ITO film using magnetron sputtering to facilitate the subsequent growth of ZnO NRs. (III) A solution was prepared by mixing HTMA and zinc nitrate hexahydrate, both with a molar concentration of 0.1 M, along with ammonia water as a precipitant at a molar concentration of 0.4 M. The film was then floated on the solution surface, with the seed layer in contact with the liquid. The solution was placed in a thermostatic oven, maintaining the temperature between 85–90 °C. After 7 h, the film was removed from the solution. Residual substances on its surface, such as ${\mathrm{Zn}}^{2+}$, ${\mathrm{NO}}_{3}^{-}$, and other ions, could potentially lead to ion exchange between the electrodes or changes in conductivity, thereby affecting charge separation and transfer. To minimize the presence of residual reaction solvents, the film was repeatedly rinsed with anhydrous ethanol and deionized water. After drying, a light yellow substance appeared on the surface, which was identified as ZnO NRs. The morphology of the ZnO NRs, as observed under a scanning electron microscope (SEM), is shown in Figure 2b. (IV) Finally, the surface of the ZnO NRs was coated with a PDMS layer using a spin coater. The spin-coating process was performed at 600 rpm for 10 s, followed by 1500 rpm for 60 s, resulting in a film thickness of approximately 80 μm. After another round of drying, the negative electrode of the TENG was manufactured.

The overall structure of the PDMS-ZnO NR electrode is shown in Figure 2a. The ZnO NRs are arranged in a vertically aligned array. The spin-coated PDMS serves several crucial functions. First, it acts as the triboelectric layer of the TENG. Second, ZnO NRs are extremely fragile and susceptible to breakage and wear; the PDMS coating provides a protective layer, ensuring a more uniform stress distribution. Third, the PDMS infiltrates the gaps between the ZnO NRs, increasing the contact area and thereby enhancing charge transfer when the ZnO NRs generate polarized charges. The thickness of the PDMS layer also plays a crucial role in the performance of the TENG. A thicker dielectric layer reduces the capacitance of the TENG, thereby increasing the open-circuit voltage. However, when the thickness becomes excessively large, the increased rigidity negatively affects surface contact efficiency, leading to a reduction in charge generation and transfer efficiency [26]. Therefore, the output performance of the PDMS-ZnO NR-based TENG is jointly determined by both PDMS and ZnO NRs. In this study, all PDMS layers were prepared using the same spin-coating parameters to ensure uniform thickness. Figure 2c shows the fabricated PDMS-ZnO NR electrode, with an uncoated PET conductive layer reserved at the bottom. The image reveals that the electrode possesses a certain degree of transparency, as the grid lines of the underlying substrate are clearly visible through the electrode.

## 3. Results and Discussion

### 3.1. Operation Mechanism

The PDMS-ZnO NRs and aluminum metal serve as the friction materials at the two ends of the TENG, generating electrical energy through the contact-separation mode. The power generation of the contact-separation TENG primarily relies on the coupling of triboelectrification and electrostatic induction.

**Figure 3:**
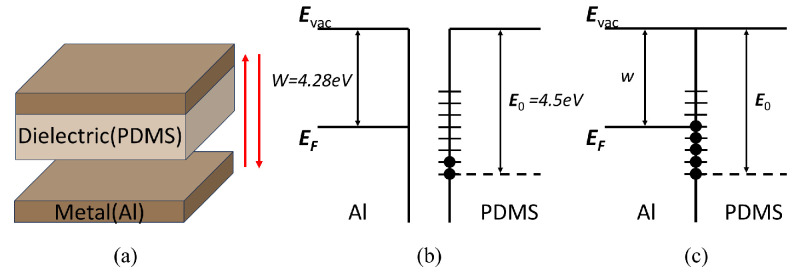
The operation mechanism of triboelectrification. (**a**) The motion model of the contact-separation TENG. (**b**) Pre-contact energy band diagram (**c**) Post-contact energy band diagram.

Triboelectrification occurs at the contact surface of the two materials, for instance, between a dielectric material and a metal. Due to the work function range of polymers being typically between 4.5 and 5.0 eV and pure aluminum being approximately 4.28 eV, the highest occupied surface state energy level, $E_{0}$, of the dielectric material PDMS is lower than the Fermi level, $E_{F}$, of aluminum. As illustrated in Figure 3, when the two materials come into contact, electrons from the aluminum flow to the surface of the PDMS to fill the surface states, thereby aligning the energy level of the PDMS with the Fermi level of the aluminum. The relationship between surface charge density and energy level difference can be expressed as follows [27]:

(1) $$E_{0}-W=\sigma_{1}ze/\varepsilon_{0}-\sigma /\bar{N_{s}\left(E\right)}e$$

where *W* represents the value of the Fermi level; σ and $\sigma_{1}$ denote the surface charge densities of PDMS and the aluminum electrode, respectively; *z* represents the distance between the aluminum and PDMS; *e* is the elementary charge; $\varepsilon_{0}$ is the vacuum permittivity; and $N_{s}\left(E\right)$ represents the surface state density.

**Figure 4:**
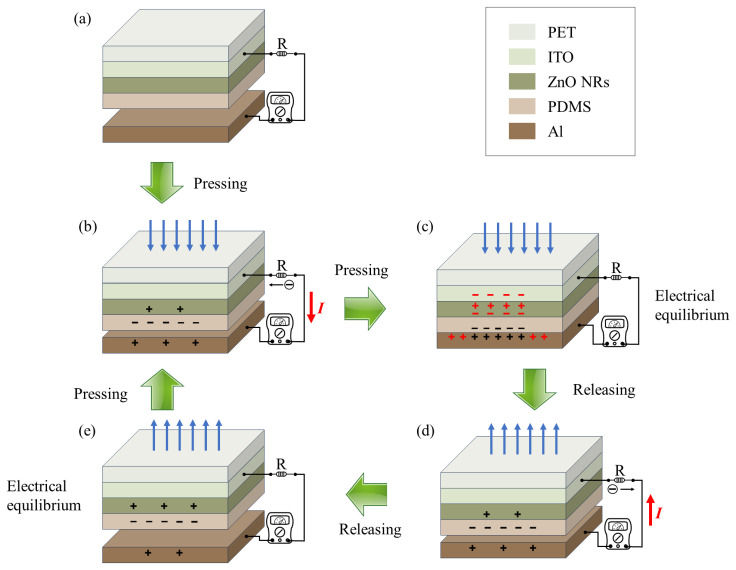
Working principle of the PDMS-ZnO NR-based TENG. (**a**) Initial state: Both electrodes are uncharged. (**b**) Contact: Charges are generated on the surfaces. (**c**) Pressing: A potential difference is created, resulting in charge flow and current generation. The red markings represent charge transfer generated by the piezoelectric effect. (**d**) Release: Static equilibrium is achieved, with no current flow. (**e**) Separation: A potential difference is generated, causing charge reflow and reverse current.

Electrostatic induction mainly arises from the relative motion between the dielectric material and the metal electrode. In this process, the piezoelectric effect of ZnO NRs plays a key role in facilitating charge transfer, as shown in Figure 4. Since PDMS is an insulating material, the surface charge of the PDMS layer tends to stabilize and can be maintained for an extended period after multiple contact-separation cycles. When the two electrodes start to approach each other, a potential difference gradually develops between them, driving negative charges from the PDMS-ZnO NR film to the aluminum electrode, thereby generating current in the external circuit. Upon contact and compression of the electrodes, the piezoelectric effect within the ZnO NRs occurs, leading to charge accumulation at the interface between the ZnO NRs and the ITO electrode. To balance the electron accumulation caused by the ZnO NRs, a positive potential develops at the metal aluminum electrode. This positive potential on the aluminum electrode is a result of the synergistic effect of the triboelectric charges and the piezoelectric effect of the ZnO NRs. When the two electrodes begin to separate, the ZnO NRs are no longer under compression, and the piezoelectric effect ceases. Meanwhile, the increasing distance between the electrodes creates a potential difference that drives the charges to flow back from the PDMS-ZnO NR film to the aluminum electrode. This compression and separation cycle generates a reciprocating alternating current, corresponding to one complete cycle of the TENG.

### 3.2. Fabrication

**Figure 5:**
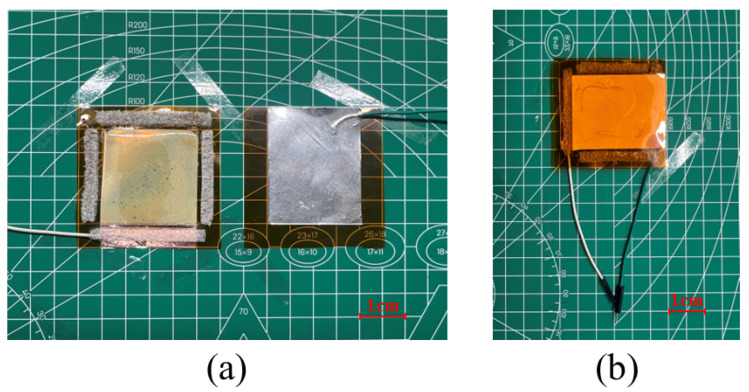
Encapsulation of TENG. (**a**) PDMS-ZnO NR triboelectric layer and Al electrode. (**b**) TENG.

The PDMS-ZnO NR film and the aluminum electrode were encapsulated using two pieces of 5 cm × 5 cm Kapton as the base substrates. The 4 cm × 4 cm PDMS-ZnO NR film and aluminum electrodes were fixed in the center of each substrate, with foam spacers of 1 mm in height used around the edges for support. The PET-ITO conductive layer was connected to the external circuit via wires, which were covered with adhesive copper foil. An insulating tape was applied over the copper foil. The connection layers of the aluminum electrode and the PDMS-ZnO NR film were offset from each other. The edges of the Kapton sheets were sealed with adhesive to complete the encapsulation. The appearance of the encapsulated device is shown in Figure 5b.

### 3.3. Electrical Performance

**Figure 6:**
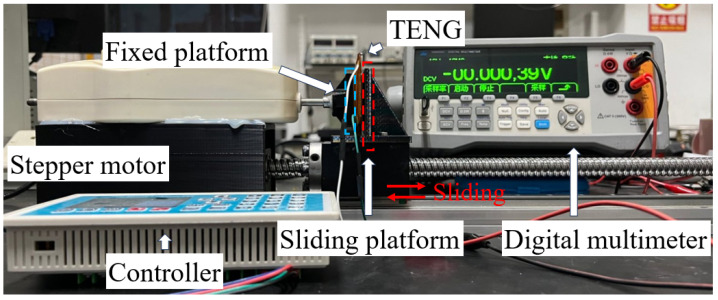
Open-circuit voltage testing platform.

To measure the electrical generation characteristics of the fabricated TENG, we designed a testing setup composed of three main components: a stepper motor, a sliding rail, a fixed platform, and a sliding platform. As shown in Figure 6, the TENG is fixed on the fixing platform, and the extrusion surface is perpendicular to the direction of the slide rail movement. A digital multimeter was used to collect and store the data.

**Figure 7:**
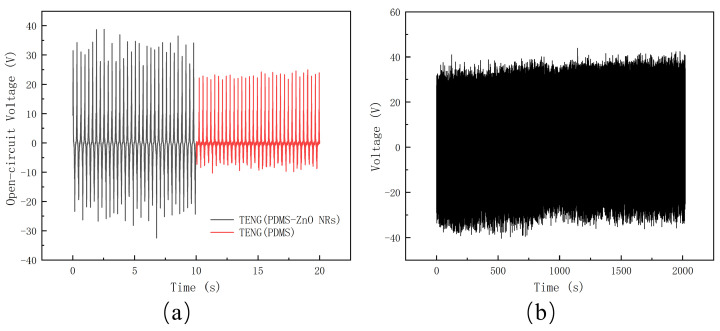
The test of open-circuit voltage. (**a**) Open-circuit voltage of the PDMS-ZnO NR-based TENG and PDMS-based TENG. (**b**) A total of 5000 cycles of testing.

The open-circuit voltage of the PDMS-ZnO NR-based TENG is shown in Figure 7a, with a maximum voltage of 39.34 V. Simultaneously, a PDMS-based TENG was fabricated using the same materials and process, with the PDMS layer having the same thickness as that in the PDMS-ZnO NR-based TENG. The maximum open-circuit voltage of the PDMS-based TENG was 23.91 V. The output voltage comparison of the two TENGs is shown in Figure 7a. The PDMS-ZnO NR-based TENG demonstrated a 64.5% increase in maximum open-circuit voltage compared to the PDMS-based TENG. Subsequently, the PDMS-ZnO NR-based TENG underwent more than 5000 cycles of testing to evaluate its durability. As shown in Figure 7b, the output of the TENG remained stable, with the output voltage maintained between 30 and 40 V.

**Figure 8:**
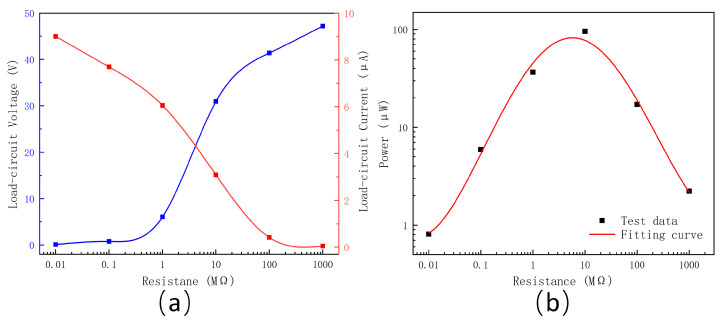
Output characteristics testing of the PDMS-ZnO NR-based TENG. (**a**) Output current and voltage under different loads. (**b**) Output power under different loads.

The load output performance of the TENG was measured by connecting a series of resistors. The output voltage and current were measured across resistors of 0.01, 0.1, 1, 10, 100, and 1000 MΩ, as shown in Figure 8a. The output voltage increased with load resistance, reaching a maximum of approximately 47.19 V. Conversely, the current decreased as the resistance increased, with a maximum value of 9 μA. The output power, as shown in Figure 8b, peaked at 82.2 μW, occurring around 5.8 MΩ. The load value corresponding to the maximum output power represents the internal resistance of the PDMS-ZnO TENG.

**Figure 9:**
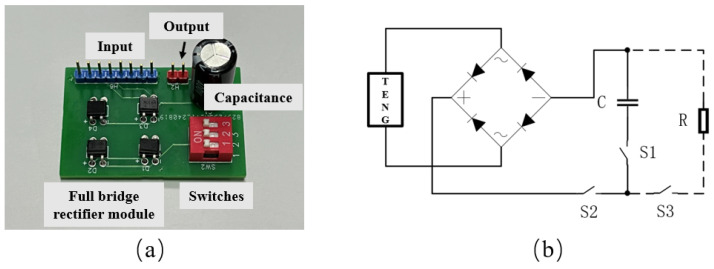
Energy-harvesting circuit. (**a**) Four-channel energy-harvesting PCB circuit. (**b**) Schematic diagram of a single-channel energy-harvesting circuit.

Since the output of the TENG is alternating current (AC), it cannot be directly used or stored. Therefore, an energy-harvesting circuit, as shown in Figure 9, which includes a rectifier bridge, switches, and a capacitor, was designed to allow the generated energy to be stored and utilized. When switches S2 and S3 are closed and S1 is open, the circuit directly powers the load. When S1 and S2 are closed and S3 is open, the circuit charges capacitor C. When S1 and S3 are closed and S2 is open, the capacitor discharges to power the load.

**Figure 10:**
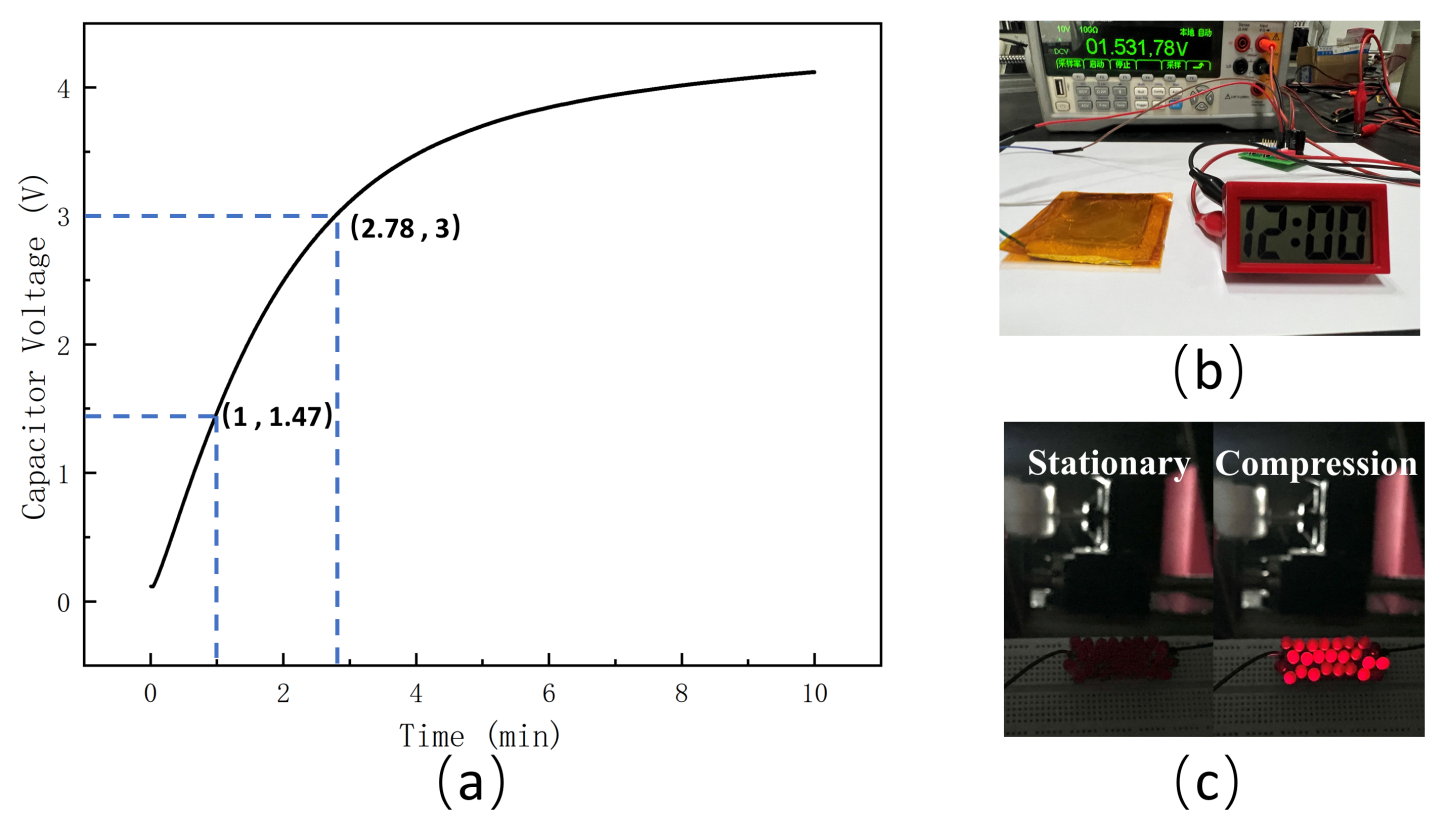
Energy-harvesting test of the PDMS-ZnO NR-based TENG. (**a**) Capacitor charging for 10 min. (**b**) Operation of the electronic watch powered by the collected energy. (**c**) Lighting the LEDs.

The charging of the capacitor in the energy-harvesting circuit using the TENG is shown in Figure 10a, with the voltage across the capacitor plotted against the charging time. The charging process lasted for 10 min, with a rapid charging rate during the first 3 min, followed by a gradual slowdown. After 8 min, the voltage stabilized near 4.2 V. The capacitor reached 1.47 V within the first minute. Subsequently, the voltage reached 3 V at 2.78 min, which is sufficient to power LED lights and other small electronic devices, as shown in Figure 10b,c.

### 3.4. Application

**Figure 11:**
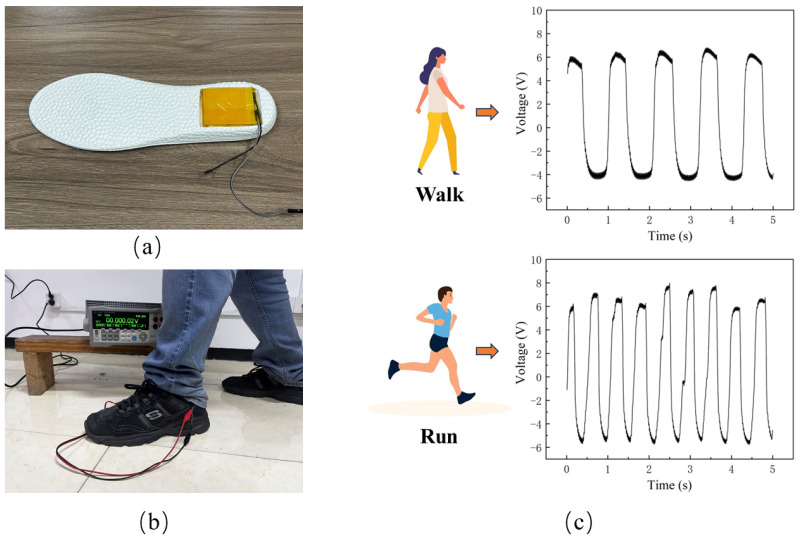
Application of the PDMS-ZnO NR-based TENG. (**a**) Integration of the PDMS-ZnO NR-based TENG into an insole. (**b**) Testing and data acquisition. (**c**) Output signals of the TENG during walking and running.

The PDMS-ZnO NR-based TENG not only harvests energy to power small electronic devices but also functions as a sensor by generating electrical signals. The fabricated TENG was integrated into footwear, where the contact and separation between the footwear and the ground during walking generate electrical signals, as shown in Figure 11a,b. The collected signals under both walking and running modes are presented in Figure 11c. Due to the cushioning effect of the insole, the recorded voltage is lower than the experimental data obtained under ideal conditions; however, a clear distinction can still be observed. In the walking mode, five peaks (or valleys) were generated within 5 s, corresponding to five steps. In the running mode, nine peaks (or valleys) appeared within the same time frame, indicating nine steps. Additionally, the voltage in the running mode is relatively higher than in the walking mode, which can be attributed to the greater impact force and the faster contact-separation process. The voltage fluctuations at the peaks (or valleys) are caused by the interaction between the TENG and the cushioning insole during compression and lift-off. Integrating TENGs into footwear and clothing enables self-powered sensing through human motion energy, aligning with the development trend of smart wearable technology.

## 4. Conclusions

In this study, to enhance the output performance of a contact-separation mode TENG, a ZnO NR film with piezoelectric properties was integrated into a PDMS film as the dielectric layer. By comparing different solution concentrations and reaction times, an optimized ZnO NR film was obtained, featuring nanorods with an average length of 7 μm and a diameter of 100 nm. A TENG was then fabricated by pairing this PDMS-ZnO NR film with an aluminum electrode, achieving a maximum open-circuit voltage of 39.34 V. Compared to a PDMS-based TENG fabricated using the same materials and process, the maximum open-circuit voltage was increased by 64.5%. The fabricated PDMS-ZnO NR-based TENG, with dimensions of 4 cm × 4 cm, exhibited a peak output power of 82.2 μW under load conditions. Subsequently, the energy generated by the TENG was harvested using a capacitor, which reached 1.47 V within 1 min and 3 V in 2.78 min, meeting the power requirements for LED lights and small sensors. Finally, the TENG was integrated into footwear, where the generated electrical signals could be used for gait analysis.

In conclusion, the theoretical analysis and experimental results confirm the significant improvement in output performance. The application of the PDMS-ZnO NR-based TENG provides a viable approach for distributed energy harvesting in compression motions. Compared to traditional mechanical electromagnetic generators, TENGs offer advantages such as compact size and high voltage. The incorporation of ZnO NR films enhances the output voltage of the original PDMS-based TENG, and its flexible material allows for seamless integration and application in various scenarios, demonstrating great potential. This study provides a qualitative analysis of the role of ZnO NRs, while the degree of improvement in TENG performance with different forms of ZnO NRs requires further investigation. The ZnO NRs used in this work were arranged in a vertical array, and when combined with 3D printing technology, it becomes possible to precisely design the microstructure and three-dimensional shape of ZnO NRs, increasing the contact area and further enhancing the electrical performance of TENGs.

## Data Availability

The original contributions presented in this study are included in the article. Further inquiries can be directed to the corresponding authors.
